# Immunohistological Evaluation of Von Willebrand Factor in the Left Atrial Endocardium and Atrial Thrombi from Cats with Cardiomyopathy

**DOI:** 10.3390/ani11051240

**Published:** 2021-04-26

**Authors:** Wan-Ching Cheng, Lois Wilkie, Tsumugi Anne Kurosawa, Melanie Dobromylskyj, Simon Lawrence Priestnall, Virginia Luis Fuentes, David J. Connolly

**Affiliations:** 1 Department of Clinical Science and Services, Royal Veterinary College, Hawkshead Lane, North Mymms, Hatfield AL9 7TA, UK; lwilkie@rvc.ac.uk (L.W.); tkurosawa@rvc.ac.uk (T.A.K.); vluisfuentes@rvc.ac.uk (V.L.F.); dconnolly@rvc.ac.uk (D.J.C.); 2 Finn Pathologists, 3c, 3 Mayflower Way, Harleston IP20 9EB, UK; mdobromylskyj@rvc.ac.uk; 3 Department of Pathobiology and Population Sciences, Royal Veterinary College, Hawkshead Lane, North Mymms, Hatfield AL9 7TA, UK; spriestnall@rvc.ac.uk

**Keywords:** von Willebrand factor, cardiomyopathy, endocardium, left atrial enlargement, immunohistochemistry, aortic thromboembolism

## Abstract

**Simple Summary:**

Disease of the heart muscle (cardiomyopathy) is very common in the domestic cat and may result in several severe outcomes. These include formation of a thrombus in the left atrium which migrates to the hindlimb cutting off the blood supply, a condition called aortic thromboembolism. Affected cats present with hindlimb paralysis and extreme pain, often requiring euthanasia on humane grounds. Several factors are known to predispose to thrombus formation, including damage to the inner cellular lining of the atrium which exposes proteins that initiates thrombosis. We studied the expression of one such protein called von Willebrand Factor in the left atrium of cats with and without cardiomyopathies and at different stages of disease severity. We found that expression increased in cats with advance disease. Obtaining a greater understanding of the role this protein has in thrombus formation may allow development of novel antithrombotic agents to help prevent this devastating consequence of feline cardiomyopathy.

**Abstract:**

Aortic thromboembolism (ATE) occurs in cats with cardiomyopathy and often results in euthanasia due to poor prognosis. However, the underlying predisposing mechanisms leading to left atrial (LA) thrombus formation are not fully characterised. von Willebrand Factor (vWF) is a marker of endothelium and shows increased expression following endothelial injury. In people with poor LA function and LA remodelling, vWF has been implicated in the development of LA thrombosis. In this study we have shown (1) the expression of endocardial vWF protein detected using immunohistofluorescence was elevated in cats with cardiomyopathy, LA enlargement (LAE) and clinical signs compared to cats with subclinical cardiomyopathy and control cats; (2) vWF was present at the periphery of microthrombi and macrothrombi within the LA where they come into contact with the LA endocardium and (3) vWF was integral to the structure of the macrothrombi retrieved from the atria. These results provide evidence for damage of the endocardial endothelium in the remodelled LA and support a role for endocardial vWF as a pro-thrombotic substrate potentially contributing to the development of ATE in cats with underlying cardiomyopathy and LAE. Results from this naturally occurring feline model may inform research into human thrombogenesis.

## 1. Introduction

Feline aortic thromboembolism (ATE) is a severe complication that can occur in 11.6–21% of cats with myocardial disease [1,2,3,4,5,6]. It happens when a thrombus usually originating in the left atrium (LA) or left atrial appendage (LAA) dislodges and obstructs a branching artery of the aorta. Common clinical signs frequently relate to the pelvic limbs and include severe pain, paraparesis, paraplegia, absence or reduced strength of femoral pulses, and cyanotic paws. ATE in cats carries a poor prognosis, with over 60% of affected cats being euthanised on presentation at first opinion practices [7].

Virchow’s triad describes three factors that contribute to venous thrombosis: blood stasis, endothelial injury, and hypercoagulability [8]. Abnormal findings associated with Virchow’s triad have been reported in cats with a variety of cardiomyopathies, which provides insights into the potential pathogenesis of feline ATE. For instance, development of spontaneous echo contrast (SEC) in the LA of cats with cardiomyopathy is associated with decreased LAA blood velocity and blood stasis [9]. A hypercoagulable state has been suggested in cats with HCM [10] and histopathologic evidence of LA endothelial damage has been observed in cats with CHF [11]. A further indication of endothelial dysfunction in cats with ATE is that plasma arginine, the precursor to nitric oxide critical for endothelial health, is lower in these cats than in than cats with cardiomyopathy alone [12]. However, the contribution of endothelial injury in the left atrial endocardium to the development of ATE has not been studied at the sub-cellular level in cats with cardiomyopathy at different stages of their disease process and only to a limited extent in humans with heart disease.

vWF is synthesised in endothelial cells and megakaryocytes and is an adhesive protein that plays an important role in thrombosis through the formation of multimers [13,14]. Endothelial vWF is either secreted into the plasma constitutively, stored in rod-shaped specialised compartments called Weibel-Palade bodies within the endothelial cells, or deposited in the subendothelium [15]. vWF of platelet origin is stored in alpha-granules within the platelet and is released upon activation. The main functions of vWF are to aid binding of platelets to exposed subendothelium and to assist in platelet aggregation via the glycoprotein Ib receptor on platelets [16,17]. The majority of plasma vWF originates from the endothelium, and in human patients the plasma concentration of vWF increases in various thrombogenic diseases reflecting endothelial injury [18]. Similarly, in cats with ATE and cardiomyopathy, the elevation in circulatory vWF has been suggested to be associated with endothelial damage [10].

In human patients with atrial distension due to a variety of cardiac conditions, expression of circulating and LA endocardial vWF protein is increased and associated with the severity of LA blood stasis and atrial remodelling and has been suggested as a predisposing factor for thrombogenesis [19,20,21,22,23].

Previous studies have also confirmed the pathogenic role of vWF in both arterial and venous thrombosis. High haemodynamic forces present in the systemic arterial system result in platelets binding to vWF through integrin αIIbβ3 for the initiation of aggregation [13,24], while patients with vWF deficiency as a result of von Willebrand disease are partly protected against arterial thrombosis [25]. vWF also contributes to thrombosis in veins, through formation of an extracellular scaffold with platelets, leukocytes, and fibrin to trap erythrocytes [26]. Mice deficient in vWF were protected from induced deep vein thrombosis [26]. Although fibrin is the best studied factor involved in trapping red blood cells (RBCs) in slow blood velocity environments [27,28,29,30], a recent in vitro study showed that vWF can bind to RBCs directly and the degree of binding is increased when blood stasis is present [31].

Currently, there is limited information regarding the contribution of endocardial vWF to thrombosis in cats with myocardial disease. However, given the pro-thrombotic action of vWF in both rodent models and human patients described above, it is likely that vWF also plays an important role in the development of LA thrombosis in cats. The aim of this study was to investigate the expression of vWF in the LA of cats at different clinical stages of myocardial disease.

## 2. Materials and Methods

### 2.1. Study Population

Cats with clinical signs related to cardiomyopathy were enrolled based on their clinical presentation of ATE, CHF or both. The control cats and those with preclinical cardiomyopathy (without clinical signs of disease) were recruited from referral and first opinion cases euthanised for a variety of reasons. Cardiomyopathy was confirmed based on characteristic cardiac structural changes on gross and histopathology using criteria previously described [32,33] (see supplementary material Table S1 for histopathological diagnostic criteria for feline cardiomyopathies) in addition to complete or point of care (POC) echocardiographic examination [34,35,36] (see Table S2 for echocardiographic diagnostic criteria for feline cardiomyopathies). Briefly for full echocardiographic examinations, all measurements were taken over three different cardiac cycles and averaged. Measurements taken included left atrium to aortic ratio (LA/Ao), maximal left ventricular freewall thickness in diastole (LVFWd), maximal interventricular septum thickness in diastole (IVSd), presence of systolic anterior motion of the mitral valve (SAM), presence of spontaneous echo contrast (SEC), and presence of a formed thrombus in the LA. SAM was defined as anterior motion of either septal or both mitral valve leaflets during systole toward the LVOT using the right parasternal long axis five chamber view on review of 2D cineloops [37]. All echocardiographic examinations were performed by a veterinary cardiology diplomate or resident under direct supervision (see supplementary material Table S5 for echocardiographic views used [38,39]). POC examinations were performed in the emergency setting by a veterinary ECC diplomate or resident in training and facilitated measurement of LA/Ao as described above and a subjective assessment of left ventricular wall thickness in diastole.

Cats were divided into four groups: (1) Control group: cats without structural and histopathological cardiac changes, (2) Subclinical group: cats with subclinical cardiomyopathy (some cats had mild LAE), (3) CHF group: cats with CHF attributable to cardiomyopathy with LAE, (4) ATE group: cats with ATE attributable to cardiomyopathy with LAE, irrespective of whether presenting with concurrent CHF.

Inclusion and exclusion criteria are listed in Table 1.

Diagnosis of ATE was based on clinical signs including acute fore/hind limb(s) paresis or plegia, loss of palpable femoral pulses, cold limbs, and cyanotic nail beds in association with myocardial disease identified by cardiac imaging [40]. CHF was diagnosed based on radiographic evidence of cardiogenic pulmonary oedema, ultrasonographical evidence of pleural effusion in association with cardiomyopathy, and left or bi-atrial enlargement. Cats with cardiomyopathy identified by cardiac imaging and gross and histopathology but showing no clinical signs of heart disease were determined to have subclinical cardiomyopathy.

### 2.2. Sample Collection

The heart was harvested within 30 min of euthanasia and flushed with slowly running tap water and each chamber was further gently flushed using a 20 mL syringe to ensure all blood was removed to facilitate optimal fixation in 10% buffered formalin. The LA samples were collected from the LA free wall. Where a formed thrombus in the LA was identified, it was carefully removed and placed into 10% buffered formalin, while an in situ thrombus in the LAA was harvested without being removed from the LAA. After gross pathological examination, sections of the left atrial free wall and cross-sections of the ventricles at the heart base, midwall, and apex were embedded in paraffin wax in a routine manner for preparation of slides for haematoxylin and eosin and Masson’s trichrome staining for histopathological examination. The LA sample blocks were used to prepare slides for immunostaining.

### 2.3. Fluorescent Immunostaining

After dewaxing, rehydration, antigen retrieval with citric acid (pH6) at 95 °C for 10 min, and blocking with 10% goat serum, slides were incubated with primary antibodies (1:500 Rabbit polyclonal IgG against vWF, Sigma, Gillingham UK; 1:25 Mouse monoclonal IgG1 against CD41 platelet marker integrin αIIb, clone B-9, Santa Cruz, CA, USA) at 4 °C overnight. An hour-long incubation of slides with 1% Bovine serum albumin/Tris-buffered saline (TBS) suspended 4′,6-diamidino-2-phenylindole (DAPI) (1:100 nuclei stain, Sigma) and secondary antibodies (conjugated with Cyanide Dyes, Cy2 1:100, Cy3 1:500, Jackson ImmunoResearch, Ely, UK) was completed at room temperature. IgG isotype control (1:400 Rabbit polyclonal IgG, Abcam, Cambridge, UK) was used to replace primary antibody against vWF to ensure specific binding. For assessment of nonspecific binding of secondary antibodies, reagent control was carried out by omitting all primary antibodies.

### 2.4. Image Analysis

All the slides were examined under a Leica DMRA2 microscope (Leica, Wetzlar, Germany) connected to a monochrome camera (AxioCam, Oberkochen, Germany) and 3 images covering the entire length of the LA endocardium were taken. The green fluorescence shown in grey scale from endocardial endothelium was selected free-hand and measured using ImageJ (https://imagej.net/Welcome, accessed on 28 February 2018). The three measurements were then averaged to give a final number representing the detected fluorescence of the endocardial sample. Exposure time was fixed for channels that detected fluorescence from DAPI and vWF for all slides. All the slides were coded so the observer (WCC) was fully blinded when analysing the images and the order in which they were viewed was randomised by a second person (DJC). The fluorescent signals from both IgG isotype control and reagent control were graphed for reference.

A Leica DM4000B with DFC550 colour microscopy camera (Leica, Wetzlar, Germany) was used for light microscopy. The microscopes and cameras were controlled using the Leica Application Suite Version 4.12 (Leica, Wetzlar, Germany).

### 2.5. Statistical Analysis

All statistical analyses were performed on GraphPad (version 8) (GraphPad, San Diego, CA, USA). Histogram and Shapiro-Wilk test were used for inspection of data distribution and normality. ANOVA test with Tukey post-hoc test and Chi-squared test with Yate correction were used to assess the difference in age and sex. Kruskal-Wallis test with Dunn’s multiple comparison test was used to compare the fluorescent intensity of labelled vWF between groups. Difference with a *p*-Value < 0.05 was considered significant.

## 3. Results

### 3.1. Animals

LA free wall samples from 39 cats were used for quantification of endocardial vWF. Thrombi were also retrieved from the LA of 3 (out of the total 39 cats) for immunohistological investigation. The LAA thrombus in situ was acquired from an extra cat that was collected later in the timeline thus not included in the quantification of endocardial vWF (Cat ATE 12 in supplementary material Tables S3 and S4). The inclusion and exclusion criteria for the 4 separate groups of cats are shown in Table 1. The cats in the control group were generally younger but this was not statistically significant (*p* = 0.137). Male cats were overrepresented in cats with cardiomyopathy (*p* = 0.009). Further demographic information about the cats used in the study are given in Table 2.

See supplementary material Tables S3 and S4 for details of clinical presentation, cardiac imaging and histopathological diagnosis for each cat used in this study. One cat in the CHF group (Cat CHF 7 in Tables S3 and S4) had been given clopidogrel prior to presentation.

### 3.2. Localisation of vWF Protein in the Left Atrial Samples

**Figure 1:**
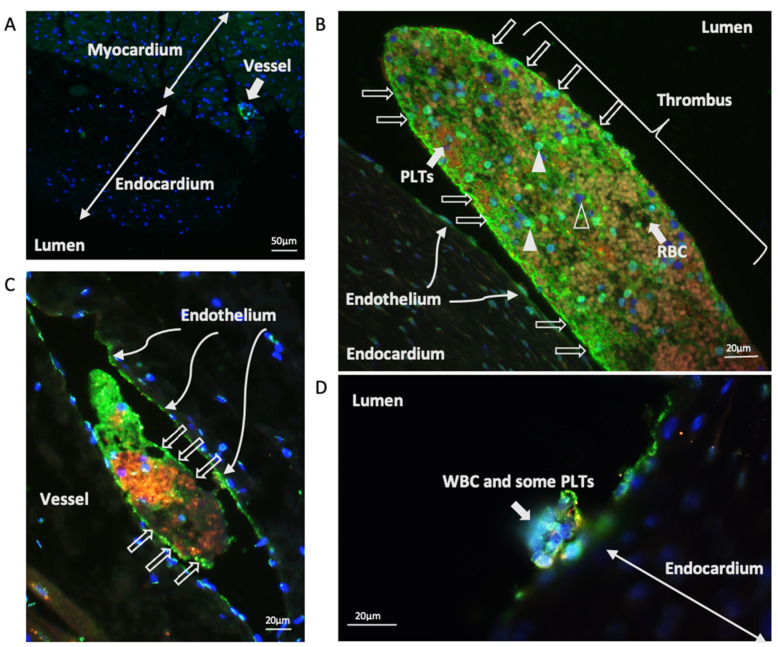
Localisation of vWF in the LA samples. (**A**) vWF localised to the endothelium of a vessel (wide arrow) in a control cat where there was minimal immunostaining of vWF at the left atrial endocardium. 100× magnification. (**B**) In this microthrombus from a cardiomyopathic cat with clinical signs, vWF appeared to form a scaffold outside (open arrows) and within the microthrombus. Red blood cells (RBC) were relatively fluorescence-lucent (thick arrow). vWF also localised to the leucocytes, (closed arrow heads) and platelets (PLTs) within the microthrombus. Some leucocytes did not immunostain for vWF (open arrow heads). Endocardial endothelial cells (thin arrows) also expressed vWF. (**C**) vWF localised to the endothelium of a vessel in a cat with cardiomyopathy and clinical signs. The intravascular microthrombus had vWF (green) encompassing the thrombus (open arrows) and contacting the vascular endothelium. Platelets and vWF co-localised in the centre of the microthrombus (orange, or yellow). (**D**) vWF (green), leucocytes, or WBC (blue) and some platelets (orange or yellow) attached on the endocardium from a cardiomyopathic cat with clinical signs. 400× magnification.

In all cats, vWF could be observed to a variable degree in the vascular endothelium (Figure 1A,C). In the majority of cats with cardiomyopathy and clinical signs (groups ATE and CHF), microthrombi, defined as thrombi only visible on microscopic examination, were identified on the vascular endothelium and occasionally on the endocardium. Conversely, microthrombi were rarely seen in the control and subclinical groups. Identifiable components of these microthrombi using immunohistofluorescence were vWF (green), platelets (reddish orange), RBC (mild autofluorescence), and leucocytes (blue) (Figure 1).

### 3.3. Quantification of Endocardial vWF Expression in Left Atrial Samples

**Figure 2:**
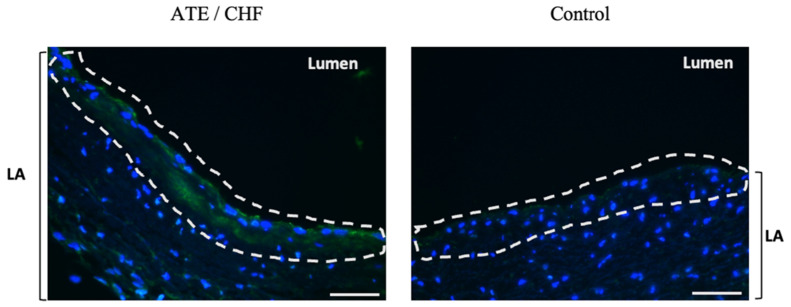
Localisation of vWF in the endocardium. Immunostaining of vWF in the endocardium. On the left, endocardial vWF (green) in a cat with cardiomyopathy and clinical signs. On the right, minimal endocardial immuostaining of vWF in a control cat. 400× magnification, Bar = 50 µm.

**Figure 3:**
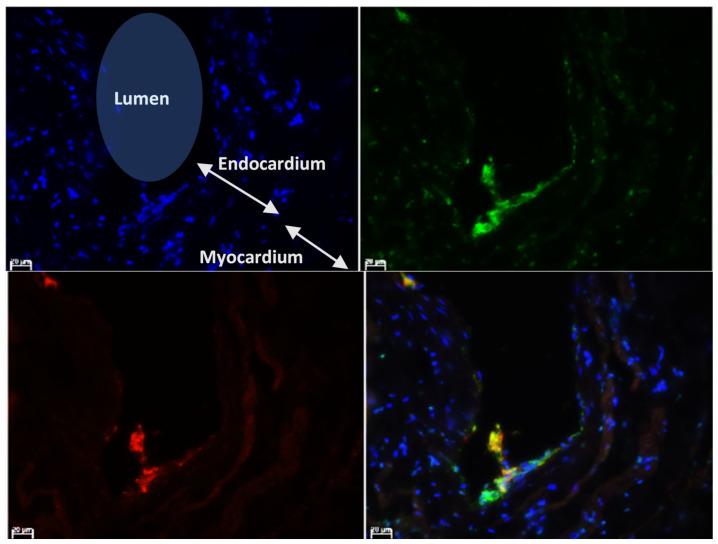
Double immunostaining for vWF and integrin αIIb. In addition to vWF, each slide was also stained for Integrin αIIb, a platelet marker, so the quantification of endocardial vWF would not include any platelets which are known to also express vWF. Images were displayed as split channels with the merged image on the bottom right. 400× magnification; bar = 20 µm; Blue—Nuclei; Green—vWF; Red—Integrin αIIb (CD41).

The intensity of vWF fluorescence at the endocardium (Figure 2) was quantified using ImageJ for comparison between groups. To avoid quantifying the fluorescence signals from platelets where vWF can also be detected, the slides were double immunostained for vWF and the platelet marker integrin αIIb (Figure 3).

**Figure 4:**
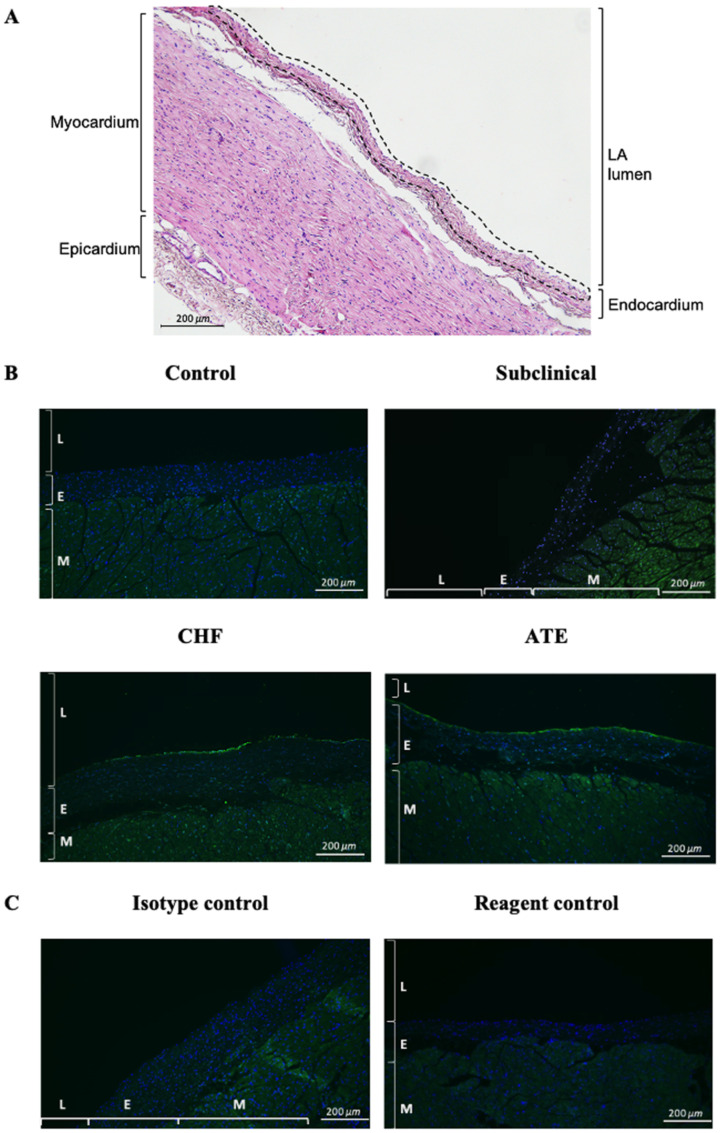
Comparison of the vWF immunostaining at the endocardium. (**A**) Image illustrating the LA anatomical structures and the region of interest where the intensity of immunofluorescence was quantified (dotted overlay). (**B**) Representative images showed the variation in immunolabelling of vWF (green) at the endocardium in the different groups of cats. (**C**) Isotype control immunostaining was performed using rabbit IgG. Reagent control was performed with the primary antibody omitted. 100× magnification; Bar = 100 µm; LA (Left atrium), L (Lumen), E (Endocardium), M (Myocardium).

**Figure 5:**
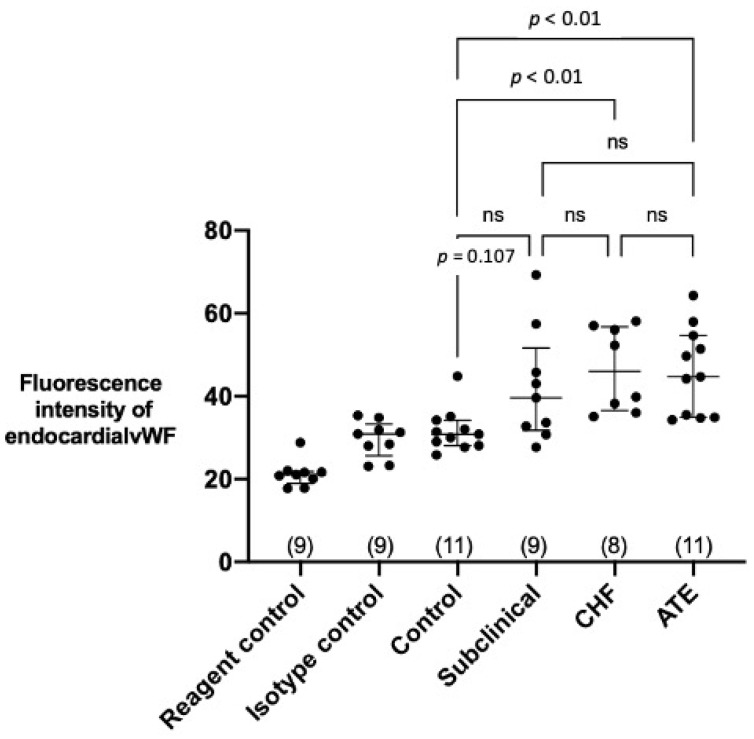
Quantification of the endocardial fluorescence from vWF: Cats with cardiomyopathy and clinical signs showed higher expression of vWF at the endocardium. The fluorescence signals detected in the ATE and CHF group were significantly higher compared to that of control group. No difference was detected between the rest of the groups. “ns” denotes non-significant. The number of cats in each group was shown in brackets. Data were analysed using Kruskal-Wallis test with Dunn’s multiple comparison test. Bars represent median and quartiles.

Representative images of vWF immunolabelling in different groups of cats are shown in Figure 4. Medians of the detected fluorescence intensity of endocardial vWF in the left atrial endocardium from the four groups from left to right as shown in Figure 5 were Control group: 30.8 (IQR 28.1–34.2), Subclinical 39.6 (IQR 31.8–59.6), CHF group: 46.0 (IQR 36.6–56.8), and ATE group: 44.7 (IQR 34.9–54.6). The fluorescence intensity was significantly higher in the ATE and CHF groups compared to the control group.

### 3.4. Characterisation of vWF in Thrombi Obtained from LA and LAA

**Figure 6:**
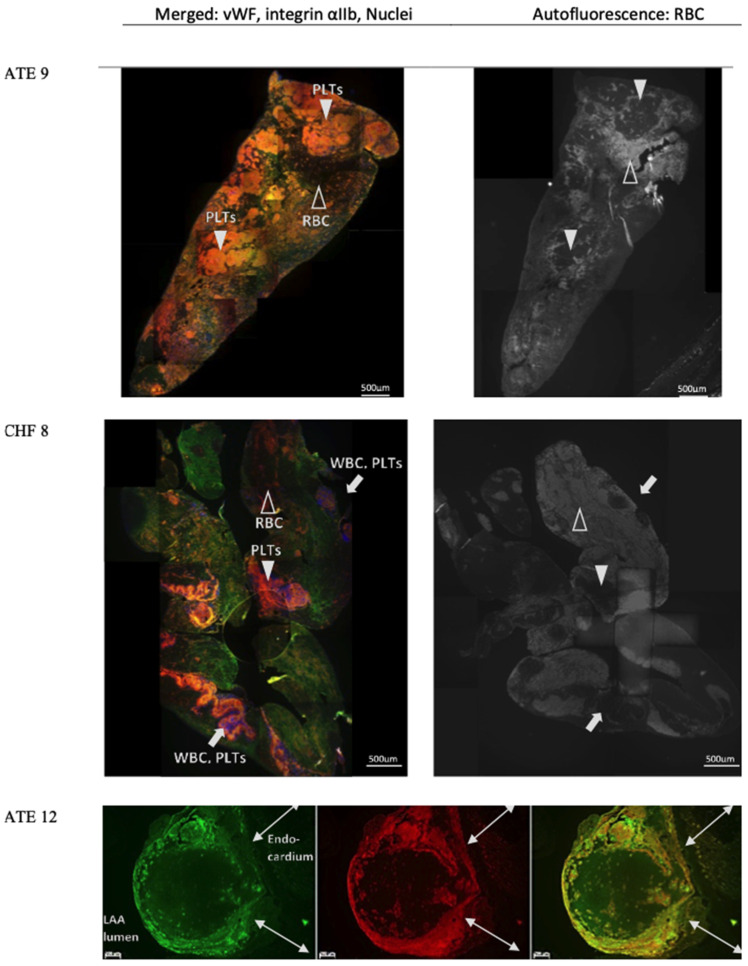
Microscopic images of thrombi immunostained for vWF and integrin αIIb: Images from Cats ATE 9, CHF 8: Images on the left shows the merged channels of vWF (green), platelet marker integrin αIIb (reddish orange) and nuclei (blue). In the merged channel, bright orange colour (closed arrow heads) represented clumps of platelets and vWF, while the RBC appeared dark and non-fluorescent (open arrow heads) compared to the immunolabelled vWF and platelets. WBCs were identified based on the blue colour of their nuclei and could be observed with platelets in clumps (arrows). Images were collated to show the whole thrombus. Images from Cat ATE 12: Split channels from left to right showed vWF in green, platelet marker integrin αIIb in red and the merged of the two channels. This thrombus remained in situ in the LAA. vWF and platelets encompassed RBC and connected to the endocardium. 50× magnification; bar = 200 µm. RBC, red blood cells; PLT, platelets; WBC, white blood cells; LAA, left atrial appendage.

**Figure 7:**
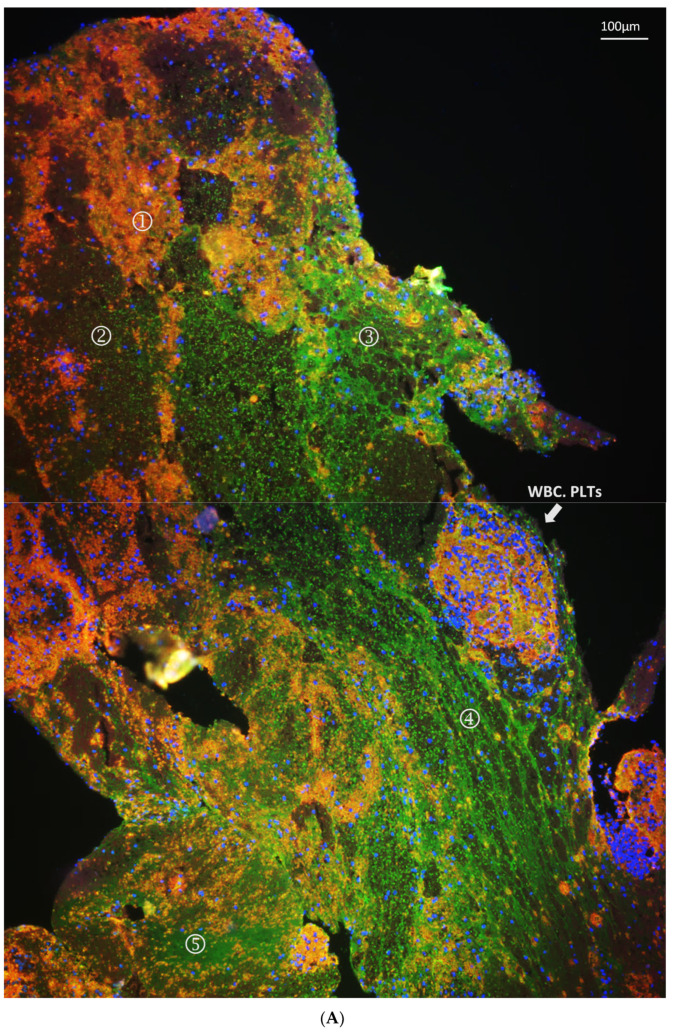

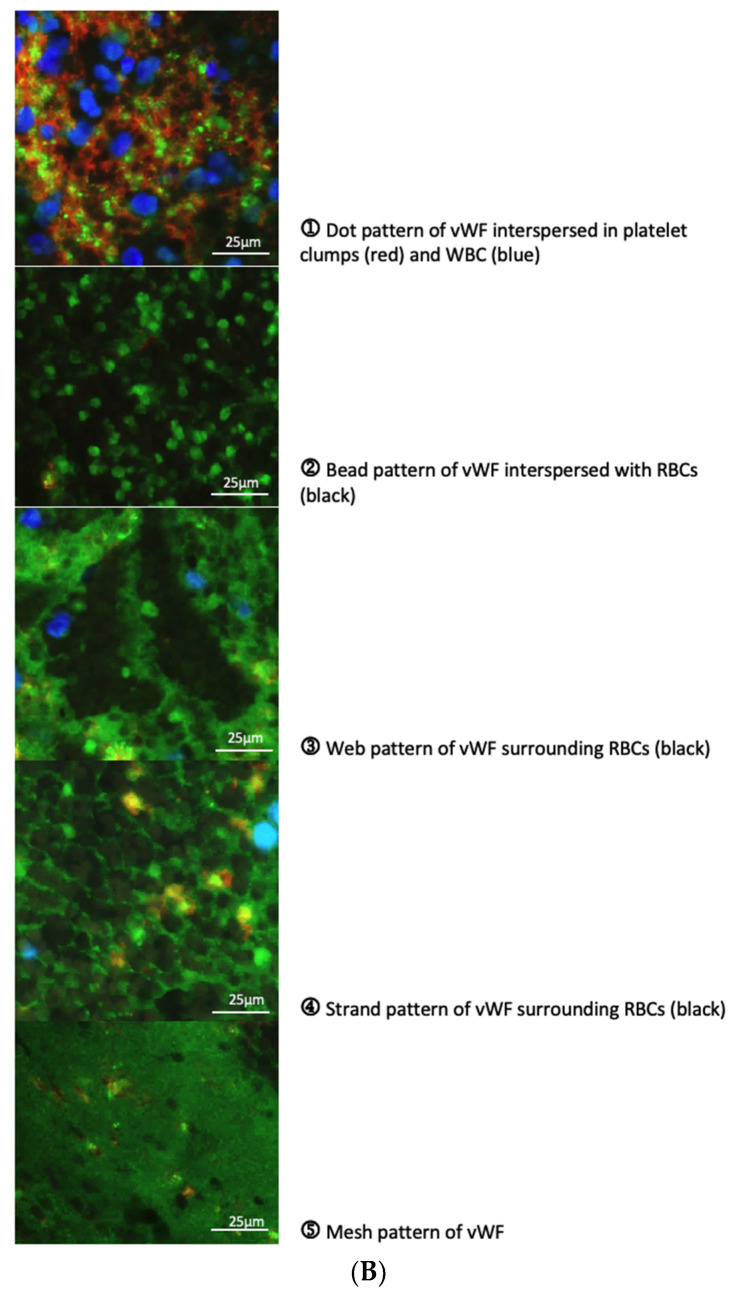
Enlarged image of the LA thrombus from Cat CHF 8. The clump of WBCs and platelets denoted by a thick white arrow in (**A**) is the same area of the thrombus from CAT CHF 8 shown in the middle left hand image of Figure 6. Different patterns of vWF staining and the variety of blood cell types in different areas of the thrombus are shown in (**B**) 1–5: 1—dots interspersed in clumps of platelets; 2—beads interspersed within RBC; 3—web surrounding RBC; 4—strands surrounding RBC; 5—mesh with minimal platelets, RBC, or WBC.

Thrombi were found in the LA or LAA in three cats (CHF 8, ATE 9, and ATE 12 in Tables S3 and S4) at postmortem. The necropsy images of the thrombi and the hearts can be found in Figure S1. The microscopic images of the thrombi, two retrieved from the LA and one remaining in situ in the LAA, are shown in Figure 6. These thrombi were immunostained for vWF (green) and platelets (reddish orange) and were counterstained with the nucleus stain (DAPI). WBC can be identified by the blue round-shaped nucleus stained by DAPI. RBC can be easily identified by their autofluorescence in the reagent controls (Figure 6, autofluorescence column) [41]. The autofluorescence of RBC was markedly weaker compared to the fluorophores bound to the antibodies used for labelling vWF, platelets and nuclei and thus appeared relatively dark in Figure 1, Figure 6, and Figure 7. The composition of each thrombi was very different in terms of relative proportions of the main components and how different components were organised. An enlarged image of the upper right quadrant of the LA thrombus from CHF 8 (Figure 6) is used as an example to show the various patterns of organisation (Figure 7A). Different patterns of vWF expression were also observed and enlarged images of these different patterns of expression are described in Figure 7B.

## 4. Discussion

Cats with underlying myocardial disease are known to be at a greater risk of thromboembolic events which frequently results in euthanasia on humane grounds [6,7]. Given the high prevalence of cardiomyopathy in cats, which for HCM is estimated at up to 17% in outbred animals and even greater in certain pedigree breeds [33], it is important to obtain a greater understanding of the left atrial remodelling process that predisposes to thrombus formation.

This is the first study to quantify and characterise vWF in the LA endocardium and LA thrombi from cats with cardiomyopathy at different stages of their disease process. LA samples from cardiomyopathic cats with LAE and clinical signs (CHF and ATE), but not those from subclinical cardiomyopathic cats had increased endocardial vWF protein compared to control cats (Figure 5), which suggests an association of endocardial vWF elevation with an advanced stage of cardiomyopathy. The immunohistological evaluation of the thrombi revealed that vWF was extensively involved in thrombus formation and organisation of both microthrombi and the three macrothrombi, which were visible on gross pathology within the LA(A) in cats CHF 8, ATE 9, and ATE 12 shown in Figure S1.

The finding that vWF localised to some vessels in the LA samples is consistent with previous report of differential expression of endothelial vWF in mice where vWF expression varied not just in different organs but even in the vessels in the same vascular tree [42]. The minimal detection of vWF in the endocardium in the control cats is similar to that in a human report where no or only minimal focal immunostaining for vWF was present in the LAA endocardium from patients without cardiac abnormalities [19,43]. It is also striking that there were no female cats in our ATE group, which is consistent with previous reports showing a male predisposition to ATE and to an increase hazard of an ATE associated death. However, the possibility of this finding being due to chance cannot be ruled out [7,44].

Importantly, our finding in cats with advanced myocardial disease are similar to two human studies where patients with hemodynamical disturbance in the LA secondary to various cardiac causes showed elevated endocardial vWF compared to the non-cardiac patients using immunohistological microscopy [19,20]. In the first of these studies, a significant increase in the expression of vWF in the endocardium of atrial appendages was identified in human patients with a variety of congenital and acquired heart diseases irrespective of the presence of atrial fibrillation. Furthermore, increased vWF expression correlated with the degree of platelet adhesion and thrombus formation [19]. In the second study involving patients with valvular and non-valvular atrial fibrillation, expression of vWF protein in the endocardium correlated with the degree of structural remodelling in the atrial wall. In addition, endocardial vWF appeared important for platelet adhesion/aggregation on the endocardium and intra-atrial thrombi formation suggesting that increased endocardial vWF may contribute to LA thrombogenesis [20]. It is recognised that inflammation per se can cause thrombosis via a VWF-mediated mechanism by elevating the level of VWF, enhancing the reactivity of VWF, and modulating the levels and activities of regulatory molecules such as the metalloprotease ADAMTS13 [45]. Heart failure is an established cause of systemic inflammation, and the increased expression of vWF in our cohort of cats with clinical signs may at least in part be related to this pro-inflammatory milieu, however the relationship between circulating concentrations of vWF and heart failure remains unclear in human studies [46,47,48] and that between endocardial vWF and inflammation is not examined.

The immunostaining of the micro- and macrothrombi supports a pivotal role for vWF in thrombus formation. Thrombosis is a multifactorial process which usually involves an abnormality in one or more of the following factors: endothelial damage and dysfunction, disturbed blood flow, and hypercoagulability. Thrombus formation begins when platelets adhere to the exposed subendothelial matrix by means of vWF that was previously deposited on the sub-endothelium by endothelial cells. Once adhered, the stimulated platelets then secrete more mediators such as ADP and thromboxane A2 that further activate additional platelets leading to propagation of the thrombus [49,50].

Traditionally, two categories of thrombus have been described based on their gross appearance and composition. A red thrombus that forms under low shear stress and is primarily composed of erythrocytes and fibrin, and a white thrombus that forms under high shear stress and contains a large number of platelets and few erythrocytes. The former mostly comprise venous thrombi where stasis of blood is evident and endothelial injury is not an absolute requirement for thrombus formation. Conversely, white thrombi are usually confined to the arterial system and form after the severe endothelial disruption such as plaque erosion or rupture as part of the atherosclerosis process [51]. In the present study, platelets were found to be an important component in the thrombi despite being formed in the environment of slow blood flow and low shear stress found in the enlarged LA. Different patterns of vWF expression in the thrombi were seen in or around particular cellular components forming different structural patterns around the same cell type, such us bead-like or web-like patterns around erythrocytes. vWF is known to fold or unfold under different conditions of shear stress and can therefore display various properties and functions as a result of exposing certain binding sites for adhesion or uncovering its cleavage site [52,53]. These various structural patterns formed by the thrombi might imply that the local environment within the LA where the thrombus developed altered over time, affecting vWF function and thereby the composition of the thrombus over its development. More research is needed to investigate how and why the feline cardiogenic thrombi showed components of both a traditional red and white thrombus.

There are a number of limitations to the study. First, the origin of the detected endocardial vWF protein in the LA samples remains undetermined. We cannot rule out the possibility that the LA endocardial vWF we detected was originally secreted by platelets or endothelia elsewhere in the body and delivered via the circulation. Second, although all the control cats had structurally normal hearts, a number did have other clinical potentially inflammatory conditions that may have affected endothelial vWF expression. However, most systemic inflammatory diseases are associated with increased level of vWF, which is contrary to the finding of low endocardial vWF in the control cats in our study. Third, in the subclinical group, four out of the nine cats had mild LAE. However, when we further sub-divided these cats into (1) subclinical with LAE, and (2) subclinical without LAE, there was no significant difference in endocardial fluorescence intensity between these subgroups and the controls. Fourth, to better evaluate the structure of the thrombi, sliced-though slides with co-immunostaining for fibrin would be helpful to gain greater understanding of thrombus organisation. However, this was beyond the scope of the study and not performed.

## 5. Conclusions

These results provide evidence for increased endocardial vWF in cats with advanced cardiomyopathy and support a potential role for endocardial vWF as a pro-thrombotic substrate.

## Figures and Tables

**Table 1:** Inclusion and exclusion criteria for case selection of cats by pathological examination.

Inclusion Criteria	**Control**	**Subclinical**
	Cats that died of non-cardiac disease with no cardiac related abnormalities detected by clinical exam, gross and histopathology ± complete or POC echocardiography.	Cats that showed no clinical signs of heart disease. Cardiomyopathy confirmed on gross and histopathology + complete or POC echocardiography.
	CHF	ATE
	Cats that showed clinical signs of CHF. Cardiomyopathy and LAE confirmed on gross pathology and histopathology + complete or POC echocardiography.	Cats that showed clinical signs compatible with ATE (irrespective of CHF). Cardiomyopathy and LAE confirmed on gross pathology and histopathology + complete or POC echocardiography.
Exclusion Criteria	For all cats with cardiomyopathy	
	Documented chronic renal disease with hypertension, hyperthyroidism, and other uncontrolled systemic diseases that may induce structural cardiac changes.	
See supplementary material Tables S1 and S2 for diagnostic criteria for cardiomyopathies by histopathology and echocardiography.		

**Table 2:** Demographics of the cats enrolled.

Group	Number of Cats	Median Age (Range) (Years)	Gender Male:Female	Breed (Number of Cats)
Control	11	2.0 (0.2–7.5)	4:7	DSH (11)
Subclinical	9	7.5 (2.5–11.0)	6:3	DSH (6) DLH (1) BSH (1) Bengal (1)
CHF	8	7.5 (2.0–17.3)	6:2	DSH (6) BSH (1) Siamese (1)
ATE	11	7 (1.8–11.0)	11:0	DSH (7) DLH (1) BSH (2) Siamese (1)

## Data Availability

The data presented in this study are available on request from the corresponding author. The data are not publicly available due to privacy issues.

## Supplementary Materials

The following are available online at https://www.mdpi.com/article/10.3390/ani11051240/s1, Figure S1: Gross appearance of the thrombi. Table S1: Pathology classification criteria for feline cardiomyopathies, Table S2: Echocardiographic classification criteria for feline cardiomyopathies, Table S3: Clinical presentation and echocardiography summary, Table S4: Signalment, echocardiographic parameters, and histopathology diagnosis, Table S5: Measurement of echocardiographic variables. References [33,36,38,39] are also cited in the supplementary materials.
