# Supplementary material for: Immunohistological Evaluation of Von Willebrand Factor in the Left Atrial Endocardium and Atrial Thrombi from Cats with Cardiomyopathy
Source: Animals (Basel). 2021 Apr 26;11(5):1240. doi: 10.3390/ani11051240 (PMC8146606; doi:10.3390/ani11051240)
Supplement: Supplementary file 1 [file animals-11-01240-s001.zip › animals-1173276-supplementary.pdf]

animals

MDPI

Supplementary Materials—Tables:

Table S1: Pathology classification criteria for feline cardiomyopathies [33].

Cardiomyopathy	Macroscopic Criteria	Microscopic criteria
HCM	+/- LVH (subjective)<br>+/- LA or biatrial dilation	Myofibre disarray ≥5% of LV<br>+/- Myocyte hypertrophy<br>+/-Interstitial fibrosis<br>+/- Intramural arteriosclerosis
HCM with LV infarct	Regional or global LV wall thinning<br>LV dilation<br>LA or biatrial dilation	Myofibre disarray ≥5% of LV<br>Replacement fibrosis<br>+/- Myocyte hypertrophy<br>+/-Interstitial fibrosis<br>+/- Intramural arteriosclerosis
eRCM	Fibrous/fibromuscular tissue bridging LV<br>OR<br>Diffuse endomyocardial fibrosis involving most of the LV	Endocardial thickening<br>Fibrosis +/- or granulation tissue extending to involve the subendocardial myocardium<br>+/- Myocyte hypertrophy<br>+/-Interstitial fibrosis<br>+/- Intramural arteriosclerosis

Table S2: Echocardiographic classification criteria for feline cardiomyopathies [33,36].

Cardiomyopathy	Diagnostic Criteria
HCM	Diastolic LV wall thickness ≥6 mm<br>+/- papillary muscle hypertrophy<br>+/- SAM<br>+/- DLVOTO
HCM with LV wall infarct	Regional LV wall thinning with hypokinesis<br>LV systolic dysfunction<br>Possible regions of diastolic LV wall thickness ≥6 mm<br>Possible LV dilation<br>LA or biatrial dilation
eRCM	Diastolic wall thickness ≤5.5 mm<br>Left atrial/biatrial dilation<br>End diastolic left ventricular diameter <20 mm<br>Fractional shortening ≥ 26%<br>Fibrous tissue bridging the left ventricular lumen

Table S3: Clinical presentation and echocardiography summary.

Identifier	Presenting Signs	Murmur	Arrhythmia	Gallop	Co-morbidities	CHF	Reason for euthanasia	Echocardiography summary	Point of care echocardiography
**Control 1**	Road trauma, tachypnoea	No	No	No	Road trauma injuries	No	Multiple trauma	-	No abnormalities identified
**Control 2**	Road trauma, tachypnoea	No	No	No	Road trauma injuries	No	Fractured pelvis and urethral rupture	-	No abnormalities identified
**Control 3**	Anuria	No	No	No	Renal disease	No	Progressive hyperkalaemia	-	No abnormalities identified
**Control 4**	Abdominal distention, diarrhoea, vacant episodes	No	No	No	Congenital PSS	No	Congenital PSS	-	No abnormalities identified
**Control 5**	Normal	No	No	No	None	No	Unsociable behaviour	-	-
**Control 6**	Ocular trauma	No	No	No	None	No	Ocular trauma and aggression	-	-
**Control 7**	Normal	No	No	No	None	No	Unsociable behaviour	-	-
**Control 8**	Normal	No	No	No	None	No	Unsociable behaviour	-	-
**Control 9**	Normal	No	No	No	None	No	Aggression, soiling in house	-	-
**Control 10**	Normal	No	No	No	None	No	Unsociable behaviour	-	-
**Control 11**	Tachypnoea, pyrexia	No	No	No	Oesophagitis, pyothorax	No	Obtundation	-	No abnormalities identified

**Subclinical 1**	III/VI holosystolic murmur investigation	Yes	No	No	None	No	Rehoming shelter cat	Mild LVH, LA WNL	-
**Subclinical 2**	Neurological forebrain signs	No	No	No	Neoplasia	No	CNS neoplasia, poor prognosis	-	LVH, LA WNL
**Subclinical 3**	Chronic diarrhoea	No	No	No	Inflammatory bowel disease	No	Severe haemorrhagic diarrhoea, poor prognosis	-	LVH, LA WNL
**Subclinical 4**	Murmur	Yes	No	No	None	No	Rehoming shelter cat	Mild LVH, LA WNL	-
**Subclinical 5**	Progressive paraparesis	No	No	No	Osteosarcoma with pulmonary metastasis	No	Poor prognosis	-	LVH, LA WNL
†**Subclinical 6**	Discharging cat bite wounds, CNS signs	No	No	No	Sepsis	No	Obtundation, poor prognosis	-	LVH, LAE
†**Subclinical 7**	Trauma	Yes	No	No	Mild anaemia, mild azotaemia	No	Pelvis fracture, spinal cord injury with no deep pain, non-ambulatory	-	LVH, LAE
†**Subclinical 8**	Murmur, inappetence	No	No	No	None	No	Rehoming shelter cat	Moderate LVH, LAE	-
†**Subclinical 9**	Murmur investigation	Yes	No	No	None	No	Owner request	Severe LVH, LAE	-
**CHF 1**	Severe dyspnoea/tachypnoea	Yes	No	Yes	None	Yes	Refractory Pulmonary oedema	CHF, Severe LVH, LAE, B-lines	-
**CHF 2**	Tachypnoea, inappetence	No	No	No	None	Yes	Financial constraints	Severe LAE, SEC, thrombus, LV areas of hypertrophy and thinning with hypokinesis, B-lines	-
**CHF 3**	Severe tachypnoea	Yes	Yes	No	None	Yes	Refractory heart failure	LVH, infarcted and thinned IVS, pleural fluid, severe LAE, RAE, RV enlargement B-lines	-
**CHF 4**	Tachypnoea	Yes	No	Yes	None	Yes	CHF, poor prognosis	LAE, LVH, pulmonary oedema B-lines	-
**CHF 5**	Severe tachypnoea	No	No	Yes	None	Yes	CHF, poor prognosis	-	Severe LVH, LAE, pulmonary oedema, pleural effusion
**CHF 6**	Collapse, tachypnoea	Yes	No	Yes	None	Yes	Cardiogenic shock	-	LVH, LAE, pulmonary oedema (B-lines)
**CHF 7**	Severe tachypnoea	No	Yes	No	None	Yes	Refractory heart failure	LAE, pleural effusion, LVH, infarcted hypomotile LVFW	-
**CHF 8**	Severe tachypnoea	No	No	No	None	Yes	Poor prognosis, financial constraints	-	LVH and LAE pulmonary oedema (B-lines), thrombus
**ATE 1**	Hind limb paresis and pain,	No	No	No	None	No	ATE, poor prognosis	-	Severe HCM, LAE, SEC
**ATE 2**	Hind limb paresis and pain tachypnoea	No	No	No	None	Yes	ATE, poor prognosis	Severe LVH and LAE, SEC, pleural fluid	-
**ATE 3**	Hind limb paresis and pain, tachypnoea	No	Yes	No	None	Yes	ATE, poor prognosis	-	LAE, SEC, pulmonary oedema (B-lines), LVH
**ATE 4**	Tachypnoea	Yes	No	No	None	Yes	ATE, poor prognosis	Severe LVH, mid-ventricular obstruction, pleural fluid SEC, Thrombus	-
**ATE 5**	Hind limb paresis and pain	No	-	-	-	-	ATE, poor prognosis	-	LAE, SEC, LVH
**ATE 6**	Hind limb paresis and pain, tachypnoea	No	No	No	None	Yes	ATE, financial constraints	LAE, LVH, pulmonary oedema (B-lines)	-
**ATE 7**	Hind limb paresis and pain, tachypnoea	-	-	-	-	-	Poor prognosis, financial constraints -	-	LAE, SEC
**ATE 8**	Tachypnoea, hind limb paresis, hypothermia	No	No	No	None	Yes	ATE, CHF, poor prognosis	-	Severe LAE, LVH, pulmonary oedema (B-lines)
**ATE 9**	Hind limb paresis and pain	Yes	No	No	None	No	ATE, poor prognosis	-	LAE, SEC, LA Thrombus, LVH
**ATE 10**	Tachypnoea, hind limb paresis, hypothermia	No	No	No	None	Yes	ATE, CHF, poor prognosis	-	Severe LAE, LVH, pulmonary oedema (B-lines), SEC Thrombus
**ATE 11**	Tachypnoea, hind limb paresis	Yes	No	No	None	Yes	ATE, CHF, poor prognosis, financial constraints	-	Severe LAE, LVH, pulmonary oedema (B-lines)

***ATE 12**	Tachycardia, tachypnoea, right forelimb paresis	No	No	Yes	None	Yes	ATE, CHF	Severe LAE, LVH, B-lines

Abbreviations: ATE, aortic thromboembolism; CHF, congestive heart failure; LAE, left atrial enlargement; RAE, right atrial enlargement; LVH, left ventricular hypertrophy, SEC, spontaneous echo contrast; IVS, interventricular septum; CNS, central nervous system; PSS, portal systemic shunt; HCM, hypertrophic cardiomyopathy. * ATE 12 the LAA thrombus in situ was acquired from this extra cat that was collected later in the timeline and not included in the quantification of endocardial vWF.

Table S4: Signalment, echocardiographic parameters, and histopathology diagnosis.

Identifier	Breed	Gender	Age (yrs)	BW (kg)	BCS	La/Ao	IVSd (mm)	LVFWd (mm)	SAM	SEC yes/no	LA Thrombus yes/no	Cardiac imaging	Histopathology
**Control 1**	DSH	F	5–8	-	5/9	1.23	-	-	-	-	-	POC	Normal
**Control 2**	DSH	MN	1.6	5.9	-	1.3	-	-	-	-	-	POC	Normal
**Control 3**	DSH	MN	5.8	4.4	5/9	1.35	-	-	-	No	No	POC	Normal
**Control 4**	DSH	F	0.2	3.9		1.4	-	-	-	-	-	POC	Normal
**Control 5**	DSH	F	<3	-	6/9	-	-	-	-	-	-	-	Normal
**Control 6**	DSH	M	4.0	-	5/9	-	-	-	-	-	-	-	Normal
**Control 7**	DSH	F	<3	-	5/9	-	-	-	-	-	-	-	Normal
**Control 8**	DSH	F	<3	-	-	-	-	-	-	-	-	-	Normal
**Control 9**	DSH	F	<3	-	5/9	-	-	-	-	-	-	-	Normal
**Control 10**	DSH	F	1.5	-	5/9	-	-	-	-	-	-	-	Normal
**Control 11**	DSH	FN	7.3	6.75	-	1.3	-	-	-	No	No	POC	Normal
**Subclinical 1**	DSH	MN	5	2.8	4/9	1.38	5.5	6.1	No	No	No	Full Echo	HCM
**Subclinical 2**	DLH	FN	10.5	5	7/9	1.4	-	-	-	No	No	POC	HCM
**Subclinical 3**	DSH	MN	4.5	3.9	3/9	1.25	-	-	-	No	No	POC	HCM
**Subclinical 4**	BSH	FN	7.5	2.8	3/9	1.38	6.0	6.2	No	No	No	Full Echo	HCM
**Subclinical 5**	DSH	MN	11	7.5	7/9	1.2	-	-	-	No	No	POC	HCM
†**Subclinical 6**	DSH	MN	8.1	4.8	3.5/9	1.6	-	-	-	No	No	POC	HCM
†**Subclinical 7**	Bengal	MN	5.4	6.4	-	1.7	-	-	-	No	No	POC	HCM
†**Subclinical 8**	DSH	MN	7.5	6.7	6/9	1.59	6.5	6.9	No	No	No	Full Echo	HCM
†**Subclinical 9**	DSH	FN	2.5	-	3/9	1.62	7.5	7.8	Yes	No	No	Full Echo	HCM
**CHF 1**	DSH	MN	13	3.2	3/9	1.76	7.5	8.3	Yes	No	No	Full Echo	HCM
**CHF 2**	DSH	MN	6	5.6	5/9	3.82	8.2	5.3	No	Yes	No	Full Echo	ES-HCM
**CHF 3**	BSH	MN	4	3.6	-	1.8	3.8	8.0	No	No	No	Full Echo	ES-HCM
**CHF 4**	Siamese	MN	8	-	-	2.1	6.4	5.9	Yes	No	No	Full Echo	eRCM
**CHF 5**	DSH	FN	8	5	4/9	2.7	-	-	-	-	-	POC	HCM
**CHF 6**	DSH	MN	2	4.0	3/9	2.2	-	-	-	-	-	POC	HCM
**CHF 7**	Russian blue	MN	7	4.0	5/9	2.5	6.5	2.8	No	No	No	Full Echo	ES- HCM
**CHF 8**	DSH	FN	11	3.3	-	1.9	-	-	-	-	Yes	POC	HCM
**ATE 1**	BSH	MN	10	5.2	-	3.1	-	-		Yes	No	POC	HCM
**ATE 2**	BSH	MN	6.9	4	-	2.28	7.5	8.4	No	Yes	No	Full Echo	HCM
**ATE 3**	DSH	MN	10	4.3	-	3.85	-	-	-	Yes	No	POC	HCM
**ATE 4**	DSH	MN	2	4.0	-	2.94	8.5	10.8	No	Yes	Yes	Full Echo	HCM
**ATE 5**	Siamese	MN	11	4.7	4/9	1.8	-	-	-	Yes	-	POC	HCM
**ATE 6**	DSH	MN	9.5	-	-	1.83	6.8	6.0	No	-	No	Full Echo	HCM
**ATE 7**	DSH	M	2	4.6	-	2.11	-	-	-	Yes	-	POC	eRCM
**ATE 8**	DSH	MN	6	-	-	4.6	-	-	-	Yes	-	POC	HCM
**ATE 9**	DLH	MN	2	3.8	-	2.03	-	-	-	Yes	Yes	POC	HCM
**ATE 10**	DSH	MN	2	3.8	-	3.28	-	-	-	Yes	Yes	POC	HCM
**ATE 11**	DSH	MN	10	-	-	2.9	-	-	-	Yes	-	POC	HCM
***ATE 12**	DSH	MN	7	-	-	2.5	-	-	-	-	Yes (LAA)	POC	HCM

Abbreviations: DSH, domestic shorthair, DLH, domestic longhair; FN, female neutered; MN, male neutered; BW, body weight; BCS, body condition score; La/Ao, ratio of LA diameter and aorta diameter; IVSd, thickness of interventricular septum in diastole; LVFWd, thickness of left ventricular free wall in diastole; SAM, systolic anterior motion of the mitral valve; SEC, spontaneous echo contrast; POC, point of care; HCM, hypertrophic cardiomyopathy; ES-HCM, end-stage HCM (defined as cats with replacement fibrosis within their left ventricular myocardium); eRCM, restrictive cardiomyopathy endomyocardial form. *ATE 12 the LAA thrombus in situ was acquired from this extra cat that was collected later in the timeline and not included in the quantification of endocardial vWF.

Table S5: Measurement of echocardiographic variables.

LA/Ao [38]	View: RPSAX at the level of the aortic valve<br>Imaging modality: 2D Imaging<br>Timing: Beginning of diastole, the first frame of aortic valve closure	Ao: From the blood-tissue interface at the midpoint of the right aortic sinus to the commissure between the noncoronary and left coronary aortic cusps<br>LA: Extension of the aortic line to the blood-tissue interface of the left atrial wall, immediately lateral to the pulmonary vein

LVFWd and IVSd [39]	View: RPLAX4ch, RPLAX5ch, and RPSAX at the level of the papillary muscles<br>Imaging modality: 2D Imaging<br>Timing: End-diastole, the last frame before the aortic valve opens (RPLAX5ch), the first frame after the mitral valve closes (RPLAX4ch), or when the left ventricular internal diameter was the largest (RPSAX)	Leading edge technique avoiding the papillary muscles or false tendon attachments<br>Average of 3 measurements from the area that measures the maximal thickness is used

RPSAX: Right parasternal short axis view. RPLAX4ch: Right parasternal long axis 4 chamber view. RPLAX5ch: Right parasternal long axis 5 chamber view.

Supplementary Materials—Figures:

Figure S1: Gross appearance of the thrombi.

ATE 9

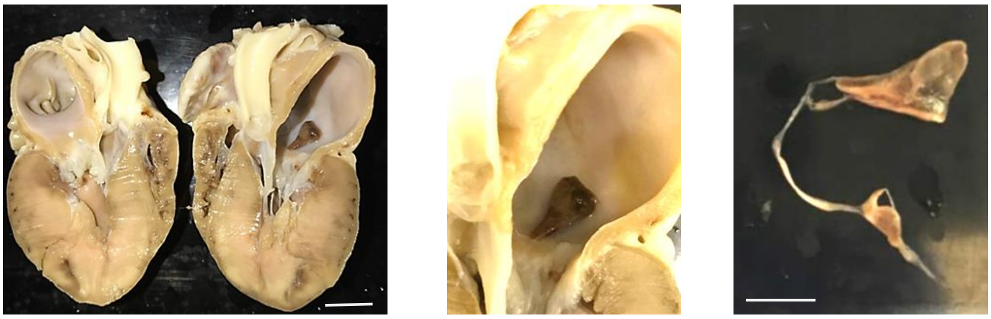

CHF 8

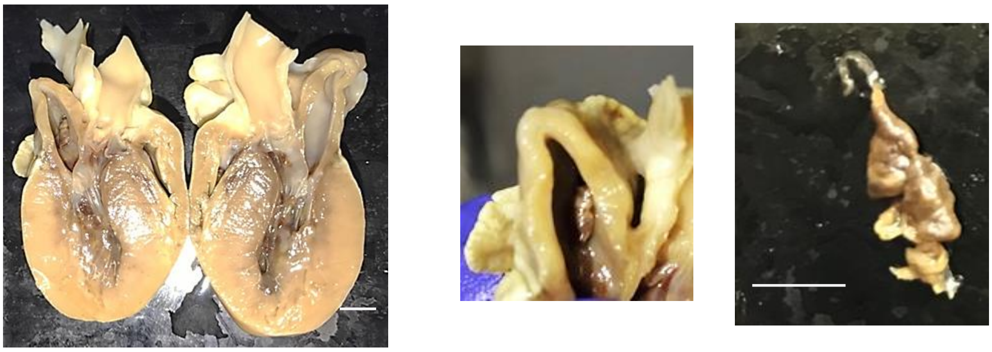

ATE 12

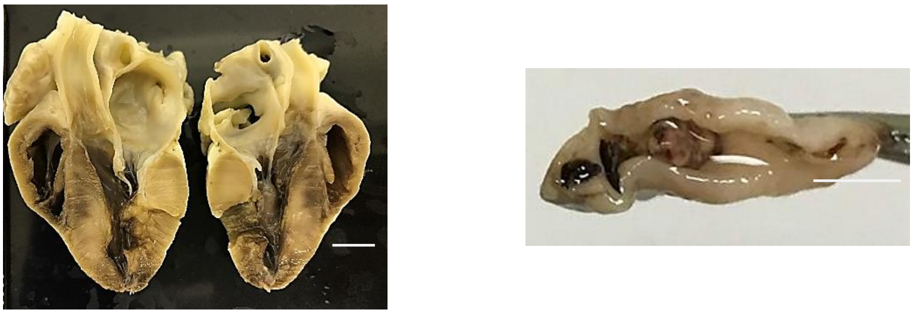

Figure S1 Gross appearance of the thrombi: The thrombi retrieved from the LA (Cat ATE 9) had both brown and white portions grossly, whereas the thrombus in the LAA (Cat ATE 12) was pale on the outside. The surface adhering to the endocardium had a relative mixed colour of the brown and white in ATE 9, while that of CHF 8 was primarily white. The LAA thrombus (ATE 12) was not taken out of the appendage so the contact phase between the thrombus, and the endocardium was preserved. *Bars next to whole heart = 1 cm; bars next to thrombus = 0.5 cm*

References

33. Wilkie, L.J. Phenotypic progression and cardiac remodelling in feline cardiomyopathies, PhD thesis, Royal Veterinary College, University of London, 2017, Hatfield, UK.
36. Côté, E.; Macdonald, K.A.; Meurs, K.M.; Sleeper, M.M. *Feline Cardiology* 1st ed.; John Wiley & Sons, Inc.: West Sussex, UK, 2011; ISBN 9781118785782.; ISBN 9781118785782.
38. Hansson, K.; Haggstrom, J.; Kvart, C.; Lord, P. LEFT ATRIAL TO AORTIC ROOT INDICES USING TWO-DIMENSIONAL AND M-MODE ECHOCARDIOGRAPHY IN CAVALIER KING CHARLES SPANIELS WITH AND WITHOUT LEFT ATRIAL ENLARGEMENT. *Vet. Radiol. Ultrasound* **2002**, *43*, 568–575, doi:10.1111/j.1740-8261.2002.tb01051.x.
39. März, I.; Wilkie, L.J.; Harrington, N.; Payne, J.R.; Muzzi, R.A.L.; Häggström, J.; Smith, K.; Fuentes, V.L. Familial cardiomyopathy in Norwegian Forest cats. *J. Feline Med. Surg.* **2014**, *17*, 681–691, doi:10.1177/1098612x14553686.
